# Guizhi-Shaoyao-Zhimu decoction alleviates gastrointestinal acute graft-vs.-host disease and suppresses FGF2/PI3K/Akt signaling: insights from network pharmacology, WGCNA, molecular docking, and animal experiment

**DOI:** 10.3389/fmed.2026.1908959

**Published:** 2026-10-23

**Authors:** Xingyu Wan, Nan Li, Xueqing Yang, Wuchen Yang, Shuiqing Liu, Xi Zhang

**Affiliations:** 1 Medical Center of Hematology, The Second Affiliated Hospital of Army Medical University, Chongqing, China; 2 Chongqing Key Laboratory of Hematology and Microenvironment, Chongqing, China; 3 Department of Hematology, Affiliated Hospital of North Sichuan Medical College, Nanchong, China; 4 Jinfeng Laboratory, Chongqing, China

**Keywords:** acute graft-vs.-host disease, molecular docking, network pharmacology, transplant immunology, WGCNA

## Abstract

**Objective:**

To evaluate the potential preventive effects of Guizhi-Shaoyao-Zhimu Decoction (GSZD) on gastrointestinal acute graft-vs.-host disease (GI-aGVHD) and explore its potential mechanisms.

**Methods:**

Bioactive compounds and putative targets of GSZD were identified by network pharmacology. aGVHD-associated genes were retrieved from public databases, and gut damage-associated genes were identified by integrating differential expression analysis and WGCNA of two public transcriptomic datasets. Protein-protein interaction network construction, enrichment analyses, and molecular docking were performed. The effects of GSZD and the potential involvement of FGF2/PI3K/Akt signaling pathway were assessed in an aGVHD mouse model.

**Results:**

A total of 183 candidate compounds and 705 GSZD targets were identified. Database mining and transcriptomic analyses identified 816 aGVHD-associated genes and 161 gut damage-associated genes. Intersection analysis revealed 109 targets shared between GSZD and aGVHD, and 11 targets shared between GSZD and aGVHD gut damage. Network and enrichment analyses highlighted AKT1, FGF2, and PI3K/Akt signaling pathway for further evaluation. Exploratory molecular docking provided predicted rankings for 31 AKT1-compound pairs and two FGF2-compound pairs. *In vivo*, compared with untreated aGVHD mice, mice treated with GSZD showed prolonged survival, reduced body weight loss, milder clinical manifestations and intestinal pathological injury, less pronounced cytokine dysfunction, improved indices of gut barrier integrity, lower FGF2 levels and PI3K/Akt phosphorylation. Exogenous FGF2 supplementation alone did not significantly worsen aGVHD, but partially reversed the protective effects of GSZD in GSZD-treated mice, accompanied by reactivation of PI3K/Akt signaling.

**Conclusion:**

GSZD showed preventive effects against GI-aGVHD in a murine aGVHD model. These effects were accompanied by attenuated inflammatory responses and reduced FGF2/PI3K/Akt signaling, suggesting that this axis may contribute to the protective effects of GSZD.

## 1 Introduction

Allogeneic hematopoietic stem cell transplantation (allo-HSCT) is a cellular therapy that infuses healthy donor-derived hematopoietic stem cells into patients following high-dose radiation or chemotherapy conditioning to reconstitute normal hematopoiesis and immune function (1, 2). For a long time, it was regarded as the only curative treatment strategy for various hematological malignancies (such as leukemia and lymphoma), and non-malignant hematological disorders (such as severe aplastic anemia and thalassemia) (3). Since the first successful allo-HSCT conducted in China in 1981, this technology has developed significantly, with annual transplant volumes exceeding 15,000 cases in 2023 (4). With the advancement of G-CSF/ATG-based haploidentical transplantation strategies, donor availability is no longer the primary constraint on allo-HSCT development, while the management of transplant-related complications has become increasingly critical to clinical success (5–7). Notably, acute graft-vs.-host disease (aGVHD), particularly its gastrointestinal (GI) form, remains the leading cause of non-relapse mortality (NRM) after allo-HSCT (8). Disruption of gut barrier facilitates bacterial and LPS translocation, driving sepsis and uncontrolled systemic inflammation. Moreover, severe diarrhea causes fluid and electrolyte imbalances alongside significant protein loss, resulting in poor clinical outcomes and treatment intolerance. Despite the availability of various prophylaxis regimens, including calcineurin inhibitor (CNI) combined with methotrexate, and post-transplant cyclophosphamide (PT-Cy), the incidence of aGVHD remains 40%–60% (9). Once aGVHD occurs, treatment options are largely limited to corticosteroid-based therapy, and patients with steroid-refractory disease generally have a poor prognosis. Therefore, advancing research on preventive and therapeutic strategies for aGVHD remains imperative to improve transplant safety and patient outcomes.

Traditional Chinese medicine (TCM), a medical system developed through long-term clinical practice of the Chinese nation, possesses a rich theoretical and empirical foundation. Currently, a series of traditional Chinese herbal formulas or TCM-derived natural compounds have been proposed for alleviating aGVHD, such as Xuebijing Injection, Baicalin, Salvianolic acid B, and Icariin (10–13). Concurrently, the advent of the network pharmacology paradigm offers robust methodological support for investigating the bioactive ingredients and molecular mechanisms of TCM formulas (14). Evolved from systems biology and poly-pharmacology, this strategy is particularly suited for the modern pharmacological analysis of TCM formulas. Based on this context, we propose Guizhi-Shaoyao-Zhimu Decoction (GSZD), a traditional Chinese herbal formula, for alleviating GI-aGVHD. This formula is composed of nine herbal medicines, including *Cinnamomi Ramulus*, *Ephedrae Herba*, *Aconiti Lateralis Radix Praeparata*, *Anemarrhenae Rhizoma*, *Paeoniae Radix Alba*, *Atractylodis Macrocephalae Rhizoma*, *Saposhnikoviae Radix*, *Zingiberis Rhizoma Recens*, and *Glycyrrhizae Radix et Rhizoma* (15). According to previous studies, it exhibits favorable anti-inflammatory and immunomodulatory effects (16, 17). For example, Ye et al. reported that GSZD alleviated rheumatoid arthritis (RA) by enriching gut *Parabacteroides goldsteinii* to deliver outer membrane vesicles that activate the Cav-1-Nrf2 axis and suppress neutrophil extracellular trap formation (16). In addition, Zhou et al. showed that GSZD inhibited the activation of NF-κB and NLRP3 inflammasome, thereby reducing the production of IL-1β, IL-6, and MCP-1 to attenuate monosodium urate crystal-induced inflammation (17). Given that the pathogenesis of GI-aGVHD involves dysregulated immune responses, excessive inflammatory cascades, and widespread cytokine storms, GSZD may hold potential for alleviating GI-aGVHD (18). Nevertheless, this formula has yet to be reported in GI-aGVHD. In this study, we explored the bioactive ingredients and molecular targets of GSZD based on network pharmacology, and identified the key targets involved in GI-aGVHD based on weighted gene co-expression network analysis (WGCNA) and multi-database mining. Subsequently, we showed that GSZD may alleviate GI-aGVHD by inhibiting FGF2/PI3K/Akt signaling pathway, based on protein-protein interaction (PPI) network analysis, Gene Ontology (GO) and Kyoto Encyclopedia of Genes and Genomes (KEGG) enrichment analyses, as well as molecular docking. Finally, we utilized a GI-dominant aGVHD mouse model to confirm the preventive efficacy and molecular mechanism of GSZD.

## 2 Materials and methods

### 2.1 Acquisition of bioactive ingredients and target prediction of GSZD

The chemical ingredients of each herb in GSZD were retrieved from the Traditional Chinese Medicine Systems Pharmacology Database and Analysis Platform (TCMSP; https://www.tcmsp-e.com/tcmsp.php; accessed on Aug 14, 2026). As TCMSP is an externally maintained database, subsequent changes in its content or accessibility may affect the exact reproducibility of compound retrieval. Compounds meeting the criteria of oral bioavailability (OB) ≥30% and drug-likeness (DL) ≥0.18 were retained as candidate bioactive compounds. Duplicate compounds shared by different herbs were merged according to their TCMSP identifiers, while the corresponding herb-compound associations were preserved. The SMILES strings of the eligible compounds were submitted to the SwissTargetPrediction database (http://www.swisstargetprediction.ch/; accessed on Aug 14, 2026), with *Homo sapiens* selected as the target species. To exclude low-confidence predictions, only protein targets with a probability score ≥0.10 were retained. The resulting genes were defined as the predicted targets of GSZD (Gene List A).

### 2.2 Acquisition of the genes related to aGVHD

The genes related to aGVHD were retrieved from 7 public databases, including GeneCards (https://www.genecards.org/), OMIM (https://www.omim.org/), DrugBank (https://go.drugbank.com/), PharmGKB (https://www.clinpgx.org/), SymMap (http://www.symmap.org/), TTD (https://ttd.idrblab.cn/), and HERB (http://herb.ac.cn/). All database searches were conducted on January 23, 2026. The detailed search strategy was provided in Supplementary Table S1. The genes obtained from each database were merged to serve as the aGVHD-related genes (Gene List B).

### 2.3 Analysis of the genes related to aGVHD gut damage by transcriptome data

Public transcriptomic datasets related to GI involvement in aGVHD were retrieved from the GEO database (https://www.ncbi.nlm.nih.gov/geo/) using the search terms “graft-vs.-host disease,” “aGVHD,” “intestinal,” “gut,” and “colon.” The database search was conducted on April 21, 2026. The inclusion criteria were as follows: (1) the species was Homo sapiens; (2) samples were derived from gut tissues; (3) the dataset included samples from patients with a definite diagnosis of GI-aGVHD and corresponding controls; (4) accessible RNA-sequencing gene-expression profiles were available; and (5) each group comprised ≥5 samples. The exclusion criteria were as follows: (1) data were derived from animal experiments or *in vitro* studies; (2) explicit grouping information was unavailable; (3) gene-annotation files were unavailable; or (4) samples were derived from tissues other than the gut.

Raw count data, transcripts per million (TPM)-normalized data, and annotation files were downloaded, and non-coding genes were excluded. Batch effects across datasets were corrected using the ComBat and ComBat-seq functions implemented in the sva R package. Raw count data were adjusted using the negative binomial-based ComBat-seq algorithm, whereas TPM data were log_2_(TPM + 1)-transformed and subsequently adjusted using the empirical Bayes-based ComBat algorithm. Correction performance was evaluated visually using principal component analysis (PCA) plots and expression-distribution boxplots. The 50 genes most strongly associated with batch effects were identified using the limma model and visualized in a clustered heatmap. Correction efficacy was further quantified by calculating the average silhouette coefficient and performing permutational multivariate analysis of variance (PERMANOVA).

Differentially expressed genes (DEGs) were identified from the count matrix using DESeq2, with the thresholds of |log2FC| ≥1 and adjusted *P* value <0.05. Adjusted *P* values were calculated using the Benjamini-Hochberg method to correct for multiple testing. In addition, WGCNA was performed based on the TPM expression matrix. Soft threshold selection was performed by evaluating the scale-free topology fit index and mean connectivity under various power parameters to ensure the scale-free topology property of the network. After the optimal power value was determined, the Pearson correlation coefficients were transformed into a weighted adjacency matrix using the power function $a_{ij}=\;|\mathrm{cor}(x_{i},x_{j}){|}^{\beta }$. Subsequently, the topological overlap matrix (TOM) and its dissimilarity measure were computed. A gene clustering dendrogram was constructed based on the TOM dissimilarity matrix, and the dynamicTreeCut algorithm was then applied to dynamically cut the dendrogram branches to identify co-expression modules. Modules with a module eigengene correlation greater than 0.75 were merged. Associations between module eigengenes (MEs) and binary aGVHD status were assessed using Pearson correlation analysis, and modules with |r| ≥0.30 and *P* < 0.05 were considered significantly associated with aGVHD. The gene significance (GS) and module membership (MM) were calculated for each gene in aGVHD-related modules. Within the aGVHD-associated modules, genes with |MM| ≥0.80 and |GS| ≥0.40 were retained as module-associated genes. These genes were subsequently intersected with the DEGs, and the overlapping genes were designated as candidate genes associated with intestinal involvement in aGVHD (Gene List C).

### 2.4 PPI network construction

Gene symbols were submitted to the STRING database (https://string-db.org/; accessed on August 17, 2026), with *Homo sapiens* selected as the species. The primary PPI network was constructed using a medium-confidence threshold (confidence ≥ 0.400). To assess the sensitivity of the results to the confidence threshold and the robustness of node prioritization, the network was reconstructed using the same input genes with a high-confidence threshold (confidence ≥ 0.700). The interaction files were exported in TSV format and visualized using Cytoscape software (version 3.10.2). Genes were ranked based on degree centrality. The robustness of the rankings was evaluated by comparing the overlap between the 10 highest-ranked genes and calculating the Spearman correlation of degree rankings obtained under the two confidence settings.

### 2.5 GO and KEGG enrichment analysis

To investigate the underlying biological implications, common genes were uploaded to the DAVID database (https://davidbioinformatics.nih.gov/; accessed on Aug 17, 2026) for GO and KEGG enrichment analyses. Enrichment results were visualized using the SRplot platform (https://www.bioinformatics.com.cn/srplot).

### 2.6 Molecular docking

3D crystal structures of AKT1 (ID: 4EKL) and FGF2 (ID: 1BFG) were downloaded in PDB format from the RCSB PDB database (https://www.rcsb.org/; accessed on May 29, 2026). The structures of ligands were downloaded in MOL2 format from the TCMSP database. Ligand structures were imported into Chem3D (v 22.0.0) and subjected to energy minimization. Protein structures were preprocessed using PyMOL software (v 3.0.3) to remove solvent molecules and native ligands. Polar hydrogens were added to the structures using AutoDockTools, and the files were converted into PDBQT format prior to docking. Molecular docking was performed as an exploratory blind-docking analysis using AutoDock Vina (version 1.2.7). The entire protein structure was encompassed by the grid box without a pre-defined binding site being specified. For each target-compound pair, the lowest-scoring pose was retained for score reporting. For each target, the two compound-target pairs with the lowest scores were selected for visualization.

### 2.7 Preparation of GSZD

GSZD herbs were purchased from the TCM Pharmacy of the First Affiliated Hospital of Army Medical University, and authenticated by two TCM professors independently. GSZD was prepared using a specific ratio of herbal components (Supplementary Table S2). The herbal mixture was soaked in 10 volumes of purified water for 30 min, rapidly brought to a boil over high heat, and then simmered for 40 min, followed by filtration through a 200-mesh sieve. The dregs were decocted again for 30 min. The combined filtrates were concentrated under reduced pressure at 60° C using a rotary evaporator to a final concentration of 2 g crude drug per mL.

### 2.8 Quality control of GSZD

The GSZD decoction prepared at a crude-herb equivalent concentration of 2 g/mL was freeze-dried to obtain a dry powder. 10 mg of the powder was extracted with 1 mL of 70% aqueous methanol to prepare a 10 mg/mL sample solution. The sample was vortexed for 30 s at 30 min intervals six times, centrifuged at 12,000 rpm for 10 min, and filtered through a 0.22 μm membrane. The filtrate was further diluted to 1 mg/mL and analyzed by UPLC-MS/MS in multiple reaction monitoring mode using a SCIEX Triple Quad 5500 mass spectrometer and a Waters ACQUITY UPLC BEH C18 column (1.8 μm, 2.1 mm × 100 mm). The reference standards used were Paeoniflorin (#B21148, Yuanye Bio-Technology), Trans-Cinnamic acid (#M043526, MREDA), Liquiritin (#V30150, Yuanye Bio-Technology), [6]-Gingerol (#G1520733, Aladdin), Atractylenolide Ⅲ (#HY-N0203, MedChemExpress), Neomangiferin (#NP01574, FUORAIN), 5-O-Methylvisammioside (#T3413, TargetMol), Timosaponin BⅡ (#V33088, InvivoChem), Benzoylmesaconine (#NP01803, FUORAIN). Chromatographic separation was performed at 40° C using 0.1% formic acid in water (mobile phase A) and 0.1% formic acid in acetonitrile (mobile phase B). Mobile phase B was increased from 5% to 95% over 9 min, maintained at 95% for 1 min, and subsequently returned to 5% for re-equilibration. The flow rate and injection volume were set at 0.35 mL/min and 2 μL, respectively. Linearity was demonstrated over 0–1,000 ng/mL (*R*^2^ > 0.995).

### 2.9 Mice

Before group allocation, healthy mice without abnormal appearance or behavior after acclimatization were included. Animals showing illness, marked body weight abnormality, or unsuccessful irradiation/transplantation before transplantation were excluded according to predefined criteria. No animals or data points were excluded from the final analyses unless otherwise stated. Six-week-old male C57BL/6 and BALB/c mice were purchased from the Vital River Laboratory Animal Technology Co. Ltd (Beijing, China), and maintained under specific pathogen-free conditions at the Experimental Animal Center of Army Medical University. Mice had free access to standard rodent chow and clean drinking water, and were housed under controlled environmental conditions (temperature: 22 ± 2° C; humidity: 50%–60%; 12 h light/dark cycle). Animal experiments were conducted in strict accordance with the National Institutes of Health Guide for the Care and Use of Laboratory Animals and the study was reported in accordance with the ARRIVE 2.0 guidelines. Animal procedures involving anesthesia or euthanasia were performed by trained personnel. For procedures requiring short-term anesthesia, including peripheral blood collection and intrarectal administration, mice were anesthetized with 3% isoflurane (#R510-22-2, RWD Life Science, China) for induction, and 1.5% isoflurane for maintenance, using a Small Animal Anesthesia Machine (#TAIJI-IE, RWD Life Science, China). Adequate anesthetic depth was confirmed by the absence of purposeful movement and response to toe pinch. After the procedure, mice were placed in a clean cage and monitored until full recovery from anesthesia. At planned endpoints or when humane endpoint criteria were met, mice were euthanized by CO_2_ inhalation using a gradual-fill method. Briefly, CO_2_ was delivered from a cylinder through a low-flow CO_2_ regulator with an adjustable flowmeter (#EP-1305, Euthanex, USA) into a transparent euthanasia chamber at 30%–70% of the chamber volume per minute. CO_2_ flow was continued for at least 1 min after respiratory arrest, and death was confirmed by the absence of respiration and heartbeat. Cervical dislocation was subsequently performed. All efforts were made to minimize animal pain and distress.

The experimental unit was an individual recipient mouse. In total, 80 BALB/c recipient mice were used for aGVHD mouse model construction, and an additional 20 C57BL/6 donor mice were used for TCD-BM and splenocyte preparation. The experiment was conducted in 2 parallel cohorts, and each cohort consisted of 5 groups (*n* = 8 per group). The sample size was determined by an *a priori* analysis performed using G*Power (version 3.1.9.7), and detailed calculations were provided in Supplementary Materials 2-Sample size power justification report. Mice in cohort 1 were utilized for monitoring survival, body weights, and clinical scores, while those in cohort 2 were utilized for histopathological staining, ELISA detection, FITC-dextran assay, and Western blotting assays. The investigator generating and implementing the randomization sequence was aware of group allocation. Because the treatment procedures differed among groups, the executor administering GSZD and FGF2 was aware of group allocation. Animals and biological samples were labeled with coded identifiers that did not reveal group assignments. Investigators responsible for survival monitoring, body-weight measurement, clinical scoring, histopathological scoring, ELISA, FITC-dextran measurements, Western blotting, and image and data analyses were blinded to group allocation. Group identities were decoded after all outcome measurements and statistical analyses had been completed. Mice were maintained under identical environmental conditions, and cage positions and experimental procedures were balanced across groups to minimize potential confounders. The animal procedures were approved by the Institutional Animal Care and Use Committee of Army Medical University (Approval No. AMUWEC20255403).

### 2.10 GI-dominant aGVHD mouse model

The mouse aGVHD was induced by transplanting major histocompatibility complex (MHC)-mismatched donor cells into recipient mice, according to previously described methods (19, 20). C57BL/6 mice were utilized as donors, and BALB/c mice were utilized as recipients. Within each cohort, recipient mice were randomly assigned in a 1:1:1:1:1 ratio to five groups, using a computer-generated random sequence, including the BMT group (negative control), the aGVHD group (disease control), the aGVHD + GSZD group, the aGVHD + FGF2 group, and the aGVHD + GSZD + FGF2 group. Recipient mice underwent 8.0 Gy ^60^Co γ-ray total body irradiation, followed by tail vein infusion of 5 × 10^6^ donor T cell-depleted bone marrow (TCD-BM) cells within 8 h. To induce aGVHD, mice in all groups except the BMT group were additionally co-infused with 5 × 10^6^ donor splenocytes.

### 2.11 Administration

Mice in the aGVHD + GSZD group and the aGVHD + GSZD + FGF2 group were administered with GSZD by oral gavage at 21.17 g/kg/day from day + 1 to day + 7 after transplantation. This dose was calculated based on body surface allometric scaling, whereby the clinical dose of 103 g/day for a 60-kg adult corresponded to a mouse-equivalent dose of 21.17 g/kg/day (*K_m_* = 37 for human, and *K_m_* = 3 for mouse) (21, 22). The day + 1 to day + 7 administration window was selected to cover the early donor T cell expansion and subsequent intestinal infiltration after transplantation, consistent with previous studies (23–25). The mice in two cohorts received GSZD from the same homogenized batch which was subjected to the UPLC-MS/MS analysis. Mice in the BMT, aGVHD, and aGVHD + FGF2 groups received an equal volume of normal saline by oral gavage according to the same schedule. For FGF2 administration, recombinant mouse FGF2 protein (#450-33-01M, Thermo Fisher) was administered locally into the intestinal lumen by intrarectal delivery based on previously described methods with minor modifications (26). Briefly, FGF2 protein was dissolved in sterile PBS containing 0.1% BSA, and mice in the aGVHD + FGF2 and aGVHD + GSZD + FGF2 groups received FGF2 at a dose of 5.0 mg/kg/day from day + 1 to day + 7 after transplantation. Under short-term isoflurane anesthesia, 100 μL of FGF2 solution was slowly administered into the distal colon using a lubricated flexible catheter inserted 3–4 cm from the anus. Mice were maintained in a head-down position for 1 min to minimize leakage. Mice in the BMT, aGVHD, and aGVHD + GSZD groups received an equal volume of PBS containing 0.1% BSA by intrarectal administration according to the same schedule.

### 2.12 Evaluation of survival, body weight and clinical scores

Survival, body weights, and aGVHD clinical scores were monitored until day + 30 after transplantation in Cohort 1. Survival status was monitored daily, while body weight and aGVHD clinical scores were monitored every 3 days. The clinical scores were assessed according to established scoring criteria (20). At the study endpoint on day + 30, remaining mice were humanely euthanized according to the CO_2_ inhalation protocol described in Section 2.9.

### 2.13 H&E staining and pathological scoring

Mice in Cohort 2 were humanely euthanized on day + 14 after transplantation by CO_2_ inhalation, as described in Section 2.9, after which small intestine and colon were collected. Tissues were washed with normal saline, and fixed in 4% paraformaldehyde (#P0099, Beyotime, China) for 24 h. Subsequently, tissues were dehydrated through graded ethanol dehydration (70%, 80%, 95%, 100%, 1 h each), cleared in xylene (2 changes, 15 min each), and embedded in paraffin. Sections (4 μm) were prepared using a rotary microtome (HM 340E, Thermo Fisher), baked at 65° C for 1 h, and stained with Hematoxylin and Eosin Staining Kit (#C0105S, Beyotime, China). Stained slides were sealed with neutral resin and visualized under a light microscope. The severity of aGVHD gut damage was scored by assessing the mononuclear cell infiltration and morphological aberrations (such as hyperplasia and crypt loss) according to previous studies, with a maximum total score of 8 (20).

### 2.14 ELISA assay

ELISA assay was performed on day + 14 after transplantation. 200 μL of peripheral blood from mice in Cohort 2 was collected after anesthesia, and allowed to clot at room temperature for 30 min, followed by centrifugation at 2,000 × g for 15 min at 4° C. The supernatant was subjected to a second centrifugation (12,000 × g, 10 min, 4° C) to remove debris, and the resulting serum was stored at −80° C until use. The serum samples were analyzed for IFN-γ (#PI507, Beyotime), TNF-α (#PT513, Beyotime), IL-6 (#PI326, Beyotime), IL-17A (#E-HSEL-M0005, Elabscience), and IL-4 (#E-HSEL-M0002, Elabscience) concentrations via ELISA according to the manufacturer's protocols. Moreover, intestinal FGF2 levels were measured using a mouse FGF2 ELISA Kit (#EMFGF2, Invitrogen) according to the manufacturer's instructions. A 2-cm segment of distal colon tissues was harvested on day + 14 after transplantation, rinsed with cold PBS, and homogenized in 500 μL of PBS containing protease inhibitors (#P1049, Beyotime). The homogenates were centrifuged at 12,000 × g for 10 min at 4° C, and the supernatants were collected for ELISA detection according to the manufacturer's protocols.

### 2.15 FITC-dextran assay

FITC-dextran assay was performed on day + 14 after transplantation. Mice were orally administered FITC-dextran (400 mg/kg; #Y240442, Beyotime) after fasting for 12 h. Peripheral blood was collected 4 h later, and serum was isolated as described in Section 2.14. Fluorescence intensity was measured at 520 nm using a microplate reader. Serum FITC-dextran concentrations were calculated based on a standard curve generated using serial dilutions of FITC-dextran. Higher serum FITC-dextran concentrations indicated increased gut permeability.

### 2.16 Western blotting assay

Western blotting was performed using colon tissues collected on day + 14 from 3 randomly selected surviving mice per group in Cohort 2. Tissues were lysed in RIPA buffer (#P0013C, Beyotime) supplemented with 2% protease and phosphatase inhibitor cocktail (#P1049, Beyotime) for 30 min on ice. Total protein concentrations were determined using a BCA assay kit (#P0010, Beyotime). Samples were separated by SDS-PAGE, transferred to PVDF membranes, and blocked with blocking buffer (#P0231, Beyotime). The PVDF membranes were incubated overnight at 4° C with primary antibodies (p-Akt, #4060, CST; p-PI3K, #17366, CST; β-actin, #8457, CST; Occludin, #ab216327, Abcam; Claudin-1, #ab307692, Abcam; ZO-1, #ab96587, Abcam). After TBST washing, the membranes were incubated with HRP-conjugated secondary IgG antibody (#7074, CST) for 2 h. Proteins were detected using ECL reagent (#P0018M, Beyotime). Following detection, the blots were stripped, washed, re-blocked, and incubated with antibodies against total Akt (#9272, CST) and PI3K (#4257, CST) for detection. Images were converted to grayscale, and bands were manually selected for intensity measurement using ImageJ. Claudin-1, Occludin, and ZO-1 were normalized to β-actin, and p-AKT and p-PI3K were normalized to their corresponding total proteins. Uncropped Western blot images were provided in the Supplementary Material.

### 2.17 Statistical analysis

IBM SPSS Statistics (version 20.0) software was used for descriptive statistics, assessment of distributional assumptions and variance homogeneity, Kaplan–Meier survival analysis with log-rank tests, and the omnibus one-way ANOVA and Kruskal–Wallis tests. R (version 4.3.3) was used for PERMANOVA, exact permutation tests, linear mixed-effects modeling, and the corresponding *post hoc* and multiple-comparison procedures. Data were presented as means ± SD unless otherwise indicated. PERMANOVA analysis was performed to assess the contributions of batch and biological condition to the overall variance following batch-effect correction of the transcriptomic data. Survival was estimated using the Kaplan–Meier method, and survival curves were compared using log-rank tests with Holm–Šídák adjustment for multiple comparisons. Clinical scores were analyzed using two-sided exact permutation tests based on the Wilcoxon rank-sum statistic at each evaluable time point, with multiple comparisons adjusted using the Holm step-down method. Body weights (normalized to each mouse's day-0 value, which was set to 100%) were analyzed using a linear mixed-effects model fitted by restricted maximum likelihood, with the Holm–Šídák method used to control the family-wise error rate. Other normally distributed data with homogeneous variances were analyzed using one-way ANOVA followed by the Holm–Šídák test, whereas non-normally distributed, ordinal, or bounded data were analyzed using the Kruskal–Wallis test followed by Dunn's test. *P* values <0.05, or adjusted *P* values <0.05 when multiple comparisons were performed, were considered statistically significant.

## 3 Results

### 3.1 Ingredients acquisition and target prediction of GSZD

To explore the pharmacodynamic material basis of GSZD, we retrieved its bioactive ingredients from the TCMSP database, yielding 1,221 natural compounds. Based on the criteria of OB ≥30% and DL ≥0.18, we further screened these compounds and identified 183 candidate molecules (Figure 1A). Subsequently, we predicted their potential target genes based on SwissTargetPrediction database, yielding 705 unique genes (Gene List A). The 15 most frequently occurring target genes were listed in Table 1.

**Figure 1:**
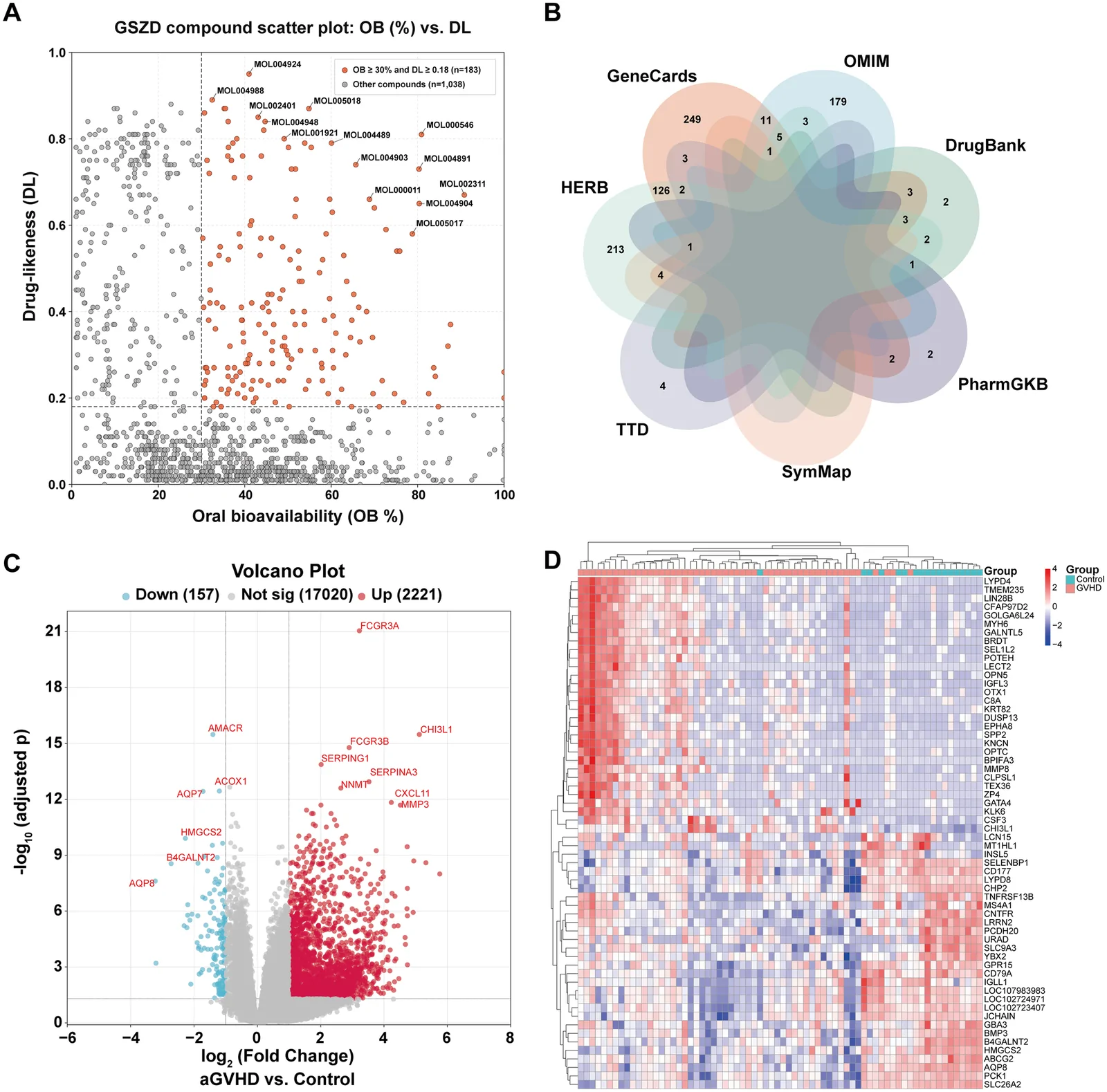
Screening of GSZD-derived compounds and identification of aGVHD-related genes. **(A)** Scatter plot of OB and drug-likeness DL values for GSZD-derived compounds. Orange dots (*n* = 183) indicated compounds meeting the screening criteria of OB ≥30% and DL ≥0.18, whereas gray dots (*n* = 1,038) indicated the remaining compounds. **(B)** Venn diagram showing the overlap of aGVHD-related genes retrieved from GeneCards, OMIM, DrugBank, PharmGKB, SymMap, TTD, and HERB. **(C)** Volcano plot of DEGs between aGVHD and control samples. Red, blue, and gray dots represented upregulated, downregulated, and non-significant genes, respectively. DEGs were defined by |log2FC| ≥1 and adjusted *P* value <0.05. **(D)** Clustering heatmap of 60 representative DEGs comprising the 30 most upregulated and 30 most downregulated genes in aGVHD samples, ranked by log_2_FC. Rows represented genes and columns represented samples.

**Table 1:** Most frequently predicted targets of GSZD-derived compounds.

Rank	Ensembl gene ID	Gene	Frequency
1	ENSG00000196396	PTPN1	123
2	ENSG00000087085	ACHE	112
3	ENSG00000140009	ESR2	111
4	ENSG00000137869	CYP19A1	108
5	ENSG00000091831	ESR1	103
6	ENSG00000012779	ALOX5	97
7	ENSG00000069535	MAOB	92
8	ENSG00000085563	ABCB1	89
9	ENSG00000074410	CA12	87
10	ENSG00000186318	BACE1	86
11	ENSG00000114200	BCHE	85
12	ENSG00000168748	CA7	84
13	ENSG00000189221	MAOA	84
14	ENSG00000133742	CA1	80
15	ENSG00000138061	CYP1B1	79

### 3.2 Acquisition of aGVHD-related targets

To investigate the molecular mechanisms of GSZD against GI-aGVHD, we first leveraged 7 databases to search for genes related to aGVHD, including GeneCards, OMIM, DrugBank, PharmGKB, SymMap, TTD, and HERB (Figure 1B). We obtained 816 distinct genes (Gene List B), which covered the vast majority of reported genes associated with aGVHD, providing a holistic profile.

### 3.3 Analysis of aGVHD gut damage-related targets

Gut damage is the primary driver of aGVHD mortality. Genes directly retrieved from public databases largely represent generic pan-immune genes, and thus fail to capture the localized gene expression changes in gut tissues. To identify genes associated with aGVHD-induced gut damage, we analyzed two available gut transcriptomic datasets from clinical aGVHD patients by combining DESeq2 and WGCNA. Following dataset integration, a total of 70 gut-tissue samples were included in the analysis, comprising 53 samples from GSE134662 (10 control and 43 aGVHD samples) and 17 samples from GSE168116 (8 control and 9 aGVHD samples). We corrected the batch effects using ComBat and ComBat-seq algorithms. Results showed that the sample clustering became decoupled from dataset origin after correction, alongside a more homogeneous gene expression profile across samples (Supplementary Figure S1). In addition, we quantitatively evaluated the correction efficacy by calculating the average silhouette coefficient and performing PERMANOVA analysis. The results confirmed a significant reduction in batch effects while preserving the biological differences between control and aGVHD groups (Supplementary Table S3).

We first identified DEGs between aGVHD and control groups by DESeq2, yielding 2,378 DEGs with 157 downregulated and 2,221 upregulated (Figure 1C). The clustered heatmap illustrated the expression profile of the top 30 upregulated and downregulated genes ranked by log_2_FC (Figure 1D). Subsequently, we employed WGCNA for further identification of key genes. Following soft threshold selection, we identified power = 20 as the optimal parameter, which ensured the scale-free topology properties while preserving robust connectivity. The fit indices and connectivity values under different powers were shown in Figures 2A,B, respectively. When power = 20, the connectivity histogram and the scale-free topology fitting plot were shown in Figures 2C,D. We established a weighted co-expression network based on this power, and initially identified 26 modules by the dynamicTreeCut algorithm (Figure 2E). Then, modules with high similarity were merged, yielding a final total of 11 modules (Figure 2E). Specifically, the Grey module contained genes that did not belong to any co-expression module, and had no interpretative value. We calculated the Pearson correlation coefficients *r* and *P* values between MEs and aGVHD, and identified 4 aGVHD-associated modules using the criteria of |*r*| ≥ 0.30 and *P* < 0.05, including Darkgrey, Black, Darkgreen, and Darkturquoise modules (Figure 2F). Furthermore, we identified module-associated genes from the 4 modules based on the criteria of |MM| ≥ 0.80 and |GS| ≥ 0.40, yielding a total of 1,312 genes (Figure 2G). Detailed information for each aGVHD-associated module was summarized in Supplementary Table S4. Finally, we identified 161 genes (Gene List C) which were closely associated with aGVHD gut damage by intersecting the module-associated genes with DEGs.

**Figure 2:**
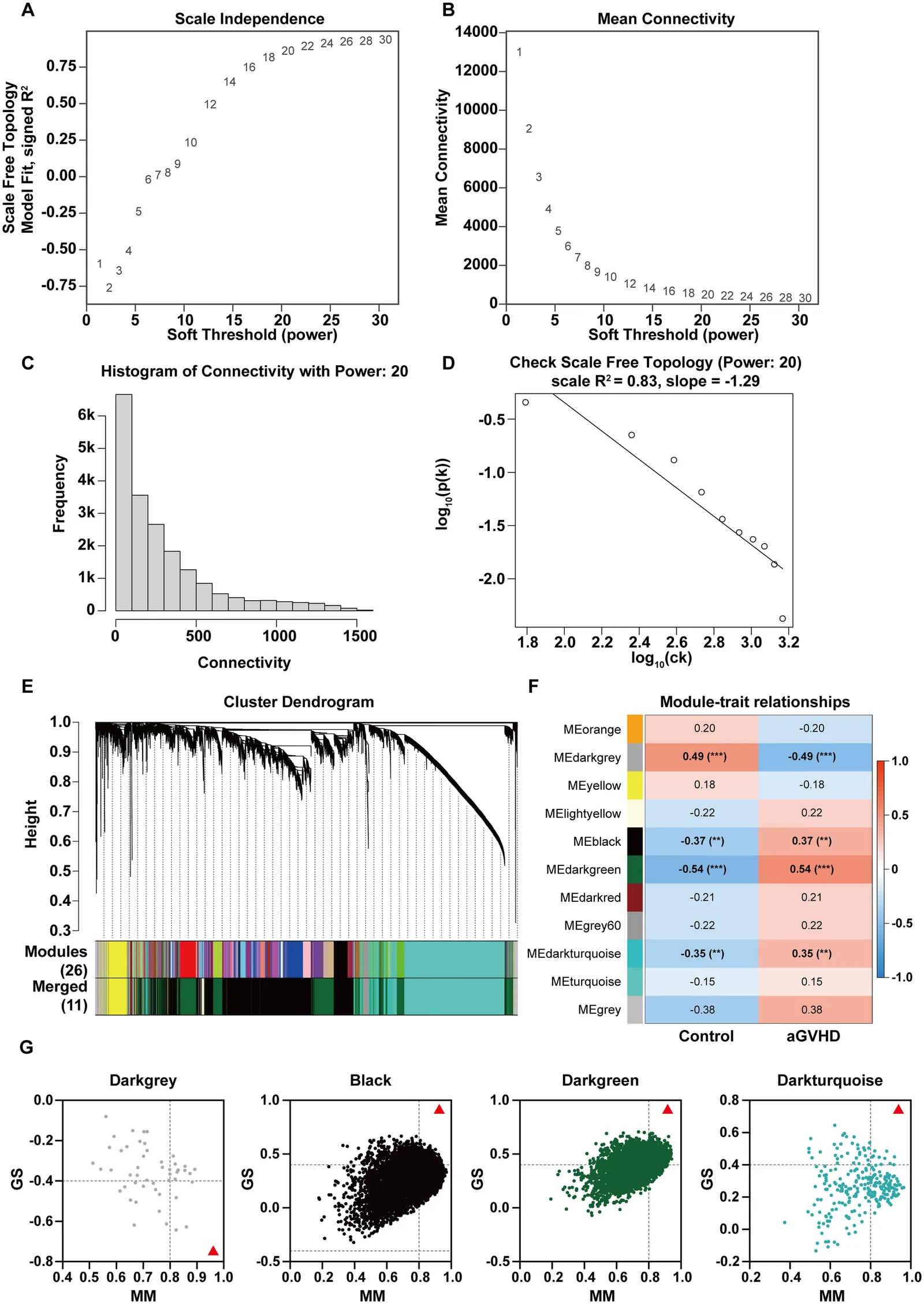
Identification of aGVHD-associated modules and genes by WGCNA. **(A,B)** The scale-free topology fit index **(A)** and mean connectivity **(B)** across candidate soft-thresholding powers. The power = 20 was selected for network construction. **(C)** Connectivity distribution at power = 20. **(D)** Scale-free topology fitting plot at power = 20 (*R*^2^ = 0.83, slope = −1.29). **(E)** The cluster dendrogram and module assignments before and after merging, showing 26 initial modules and 11 merged modules. **(F)** Heatmap of Pearson correlations between MEs and phenotypes. The numbers indicated correlation coefficients. Four aGVHD-associated modules were identified using |*r*| ≥0.30 and *P* < 0.05. *P < 0.05, **P < 0.01, ***P < 0.001. **(G)** Scatter plots of GS vs. MM in the four aGVHD-associated modules. Dashed lines indicated the thresholds of |MM| ≥0.80 and |GS| ≥0.40, and red ▴ indicated the regions containing genes meeting the criteria.

### 3.4 Network analysis of common genes between formula targets and disease genes

Based on the above results, we finally identified 816 aGVHD-related genes (Gene List B) and 161 aGVHD gut damage-related genes (Gene List C). To further investigate the potential mechanisms by which GSZD alleviates aGVHD and associated gut damage, we intersected the predicted GSZD target genes (Gene List A) with Gene List B and Gene List C, yielding 109 and 11 common genes, respectively (Figures 3A,B). Moreover, we constructed an association network to visualize the relationship between herbs, compounds, and genes (Figure 3C). The 109 common genes shared between Gene List A and B were subsequently subjected to PPI network analysis, to identify highly connected candidate genes. A primary network was constructed using a medium-confidence threshold (confidence ≥ 0.400). Results showed that the 10 highest-ranked genes were IL6, TNF, IL1B, AKT1, STAT3, PIK3CD, MTOR, NFKB1, TLR4, and BCL2, based on degree centrality (Table 2, Figure 3D). Collectively, these genes represent a core network involving inflammatory cytokine signaling, innate immune activation, cell survival, and tissue damage/remodeling, which may contribute to the pathogenesis of aGVHD. In addition, the network was reconstructed using a high-confidence threshold (confidence ≥ 0.700), as a sensitivity analysis to assess the robustness of the node rankings. Eight of the 10 highest-ranked genes were retained, and the overall degree rankings were strongly correlated across the two confidence settings (Spearman's *ρ* = 0.912, *P* < 0.001), supporting the robustness of the node prioritization (Figure 3E, Supplementary Table S5).

**Figure 3:**
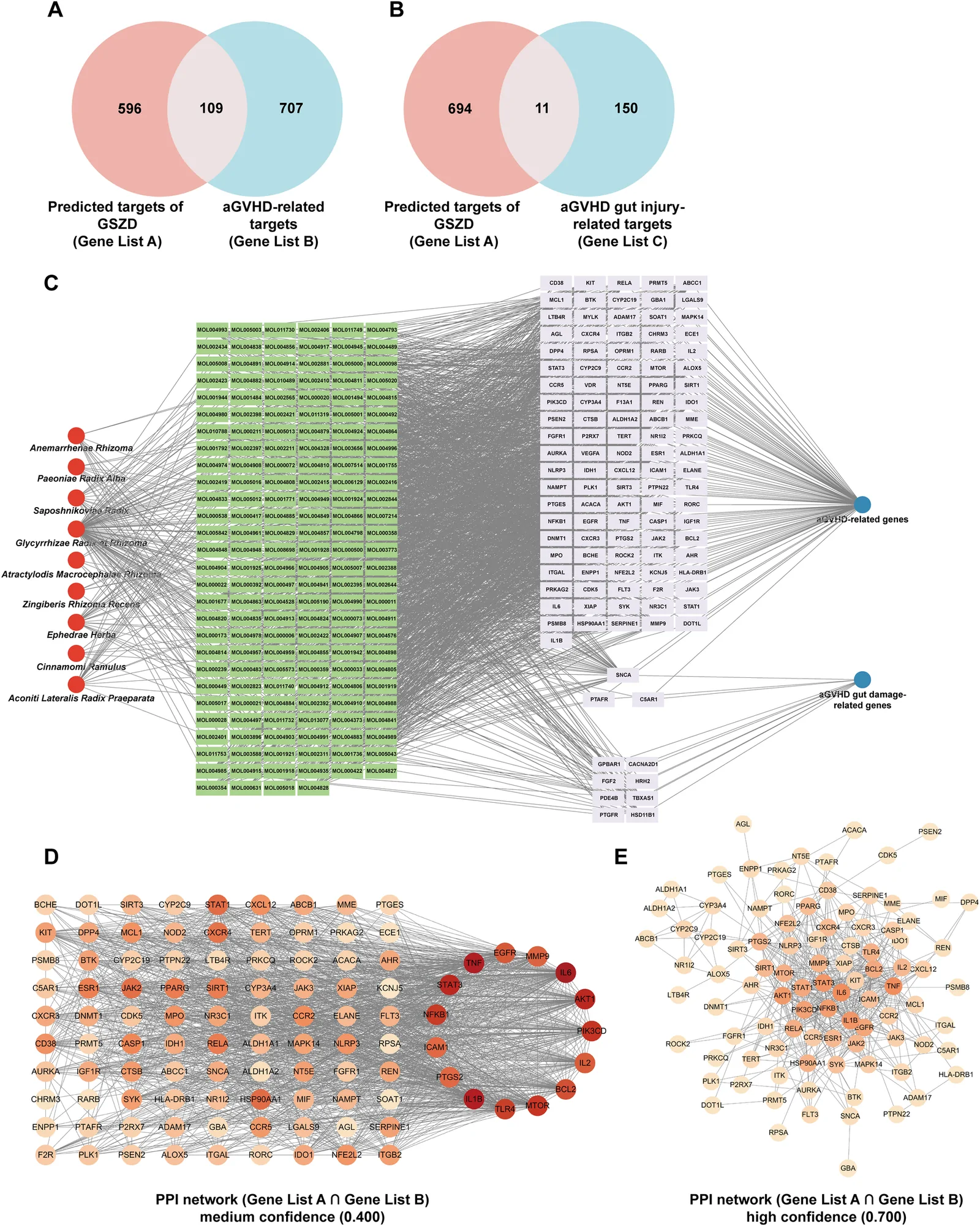
Network analyses of GSZD targets. **(A)** Venn diagram showing 109 genes shared between the predicted targets of GSZD (Gene List A) and aGVHD-related targets (Gene List B). **(B)** Venn diagram showing 11 genes shared between Gene List A and aGVHD gut injury-related targets (Gene List C). **(C)** Herb-compound-gene association network showing the relationships among the constituent herbs of GSZD, screened compounds, and shared genes. Only genes shared between the predicted GSZD targets and either Gene List B or C, together with their associated compounds, were displayed. **(D,E)** PPI networks of the 109 genes shared between Gene List A and B, constructed using medium (0.400) and high (0.700) confidence thresholds, respectively. Nodes represented proteins, and edges represented protein interactions. Darker node colors indicated higher connectivity.

**Table 2:** Top genes ranked by degree centrality in the PPI network.

Rank	Gene	Degree centrality
1	IL6	84
2	TNF	80
3	IL1B	78
4	AKT1	73
5	STAT3	73
6	PIK3CD	68
7	MTOR	67
8	NFKB1	66
9	TLR4	65
10	BCL2	60

The 11 genes shared between Gene Lists A and C were FGF2, SNCA, TBXAS1, HSD11B1, PTAFR, PTGFR, GPBAR1, HRH2, C5AR1, CACNA2D1, and PDE4B. According to previous studies, these genes were primarily involved in growth factor signaling and tissue remodeling, lipid mediator and GPCR signaling, glucocorticoid metabolism, complement activation, bile acid sensing, and cAMP/Ca2 + -dependent neuroimmune regulation, suggesting their potential involvement in intestinal inflammation and tissue homeostasis. Given the limited size of this gene set, PPI network analysis was not performed because its topological interpretation would be unreliable.

### 3.5 GO and KEGG analyses

We performed GO and KEGG enrichment analyses to comprehensively explore the biological functions and related signaling pathways of the common genes between GSZD target genes and disease-associated genes. Our results revealed that the common genes between Gene List A and Gene List B were mainly enriched in terms “Signal transduction”, “Inflammatory response”, “Immune response”, “Positive regulation of gene expression”, and “Apoptotic process” for biological process (BP); “Cytoplasm”, “Plasma membrane”, “Cytosol”, “Membrane”, and “Nucleus” for cellular component (CC); “Protein binding”, “Identical protein binding”, “ATP binding”, “Metal ion binding”, and “Protein kinase activity” for molecular function (MF) (Figure 4A). KEGG enrichment analysis showed that they were significantly enriched in pathways such as “PI3K-Akt signaling pathway”, “NOD-like receptor signaling pathway”, “Th17 cell differentiation”, “Chemokine signaling pathway”, “C-type lectin receptor signaling pathway”, “TNF signaling pathway”, “Neutrophil extracellular trap formation”, “NF-kappa B signaling pathway”, “JAK-STAT signaling pathway”, and “MAPK signaling pathway” (Figure 4B).

**Figure 4:**
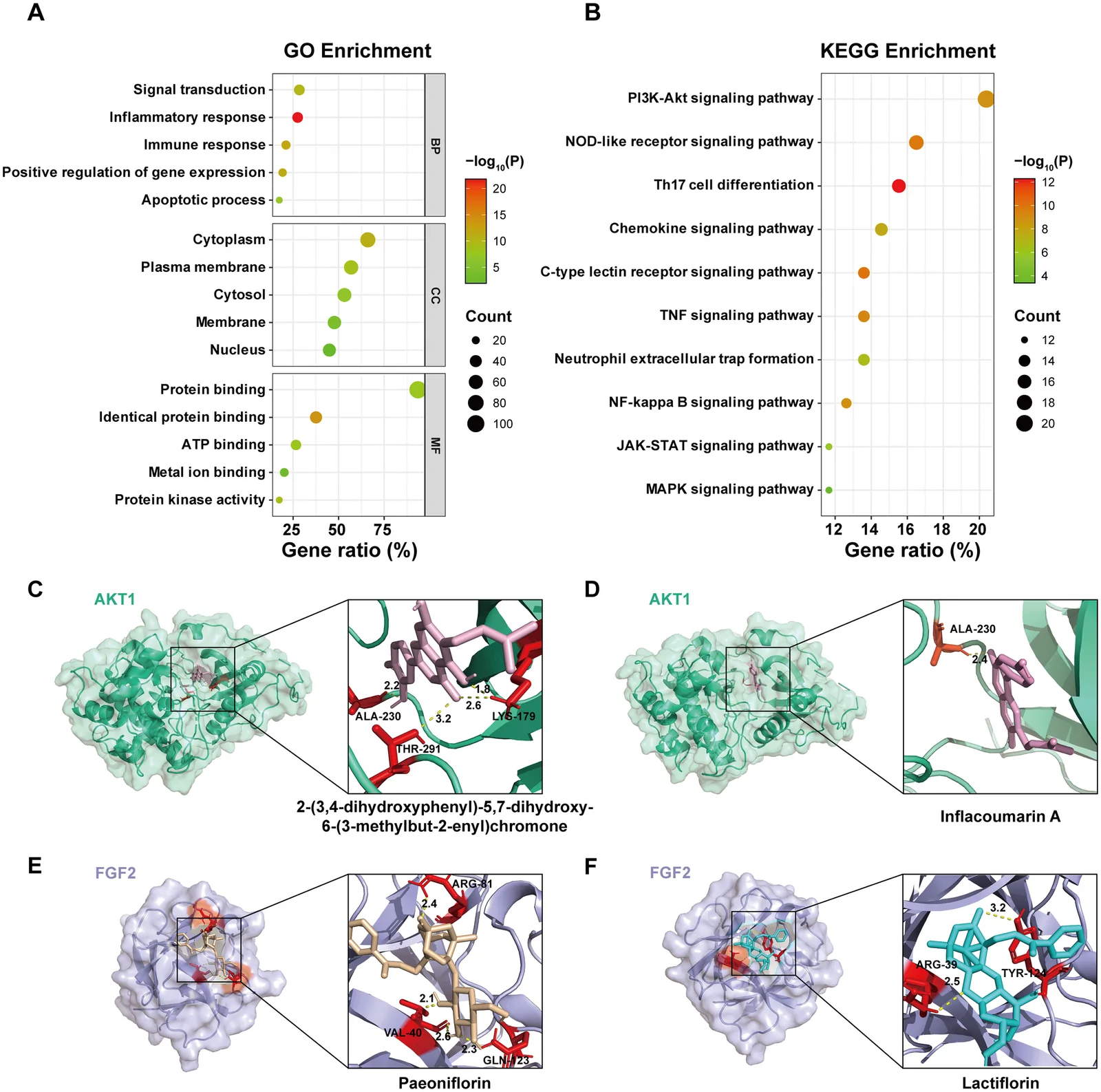
Functional enrichment and molecular docking analyses. **(A,B)** GO and KEGG enrichment analyses of the 109 shared genes between the predicted GSZD targets (Gene List A) and aGVHD-related targets (Gene List B). **(C,D)** Predicted docking poses of AKT1 with 2-(3,4-dihydroxyphenyl)-5,7-dihydroxy-6-(3-methylbut-2-enyl)chromone **(C)** and Inflacoumarin A **(D)**, respectively. **(E,F)** Predicted docking poses of FGF2 with Paeoniflorin and Lactiflorin, respectively. Enlarged views showed the modeled placement of the compounds relative to nearby amino acid residues in the selected docking poses; dashed lines mark software-predicted hydrogen bonds.

As for the common genes between Gene List A and Gene List C, GO_BP terms such as “G protein-coupled receptor signaling pathway”, “Chemotaxis”, and “Positive regulation of ERK1 and ERK2 cascade”, GO_CC terms such as “Plasma membrane”, GO_MF terms such as “G protein-coupled receptor activity”, and KEGG terms such as “Calcium signaling pathway” and “Neuroactive ligand-receptor interaction” were significantly enriched (Supplementary Figure S2).

### 3.6 Molecular docking results

We focused on PI3K/Akt signaling because AKT1 was a highly connected node in the PPI network of genes shared between the predicted GSZD targets and aGVHD-associated genes. Specifically, AKT1 ranked fourth by degree centrality (degree = 73) in this network (Table 2, Figure 3D), and remained among the top 10 at the higher confidence threshold (Supplementary Table S5). KEGG enrichment also identified PI3K/Akt signaling among the significantly enriched pathways (Figure 4B). Previous studies have shown that aberrant activation of PI3K-Akt signaling was involved in the alloreactive T cell responses and aGVHD development (27–29). In addition, FGF2, an upstream activator of PI3K/Akt signaling, was identified among the genes shared between Gene List A and C (30–32). Therefore, we selected AKT1 and FGF2 for exploratory molecular docking.

All GSZD-derived compounds predicted to target either AKT1 or FGF2 were included in the exploratory docking analysis, yielding 33 target-compound pairs (31 for AKT1 and two for FGF2). For AKT1, docking scores ranged from −9.2 to −6.2 kcal/mol. For FGF2, the scores were −6.8 kcal/mol for Lactiflorin and −6.6 kcal/mol for Paeoniflorin (Table 3). The two lowest scoring pairs for each target were selected for pose visualization (Figures 4C–F). These results provided a preliminary basis for prioritizing compounds within each target for further investigation, but did not establish evidence of physical binding or functional inhibition.

**Table 3:** Molecular docking results.

Protein	Ligand ID	Ligand name	Minimum binding energy (kcal/mol)
AKT1	MOL000006	Luteolin	−8.6
AKT1	MOL000073	ent-Epicatechin	−8.0
AKT1	MOL000098	Quercetin	−8.7
AKT1	MOL000354	Isorhamnetin	−8.7
AKT1	MOL000422	Kaempferol	−8.0
AKT1	MOL000492	(+)-Catechin	−8.5
AKT1	MOL000500	Vestitol	−7.7
AKT1	MOL001736	(−)-Taxifolin	−8.2
AKT1	MOL002398	Karanjin	−8.6
AKT1	MOL002823	Herbacetin	−8.0
AKT1	MOL002881	Diosmetin	−8.3
AKT1	MOL003656	Lupiwighteone	−8.4
AKT1	MOL004328	Naringenin	−7.6
AKT1	MOL004373	Anhydroicaritin	−8.6
AKT1	MOL004576	Taxifolin	−8.0
AKT1	MOL004798	Delphinidin	−8.0
AKT1	MOL004828	Glepidotin A	−8.8
AKT1	MOL004835	Glypallichalcone	−8.1
AKT1	MOL004841	Licochalcone B	−8.0
AKT1	MOL004857	Gancaonin B	−8.4
AKT1	MOL004863	3-(3,4-dihydroxyphenyl)-5,7-dihydroxy-8-(3-methylbut-2-enyl)chromone	−8.6
AKT1	MOL004864	5,7-dihydroxy-3-(4-methoxyphenyl)-8-(3-methylbut-2-enyl)chromone	−8.7
AKT1	MOL004866	2-(3,4-dihydroxyphenyl)-5,7-dihydroxy-6-(3-methylbut-2-enyl) chromone	−9.2
AKT1	MOL004949	Isolicoflavonol	−8.9
AKT1	MOL004961	Quercetin der.	−8.1
AKT1	MOL004980	Inflacoumarin A	−9.1
AKT1	MOL005008	Glycyrrhiza flavonol A	−8.7
AKT1	MOL005190	Eriodictyol	−8.8
AKT1	MOL006129	6-Methylgingediacetate2	−6.2
AKT1	MOL010489	Resivit	−8.3
AKT1	MOL010788	Leucopelargonidin	−8.3
FGF2	MOL001921	Lactiflorin	−6.8
FGF2	MOL001924	Paeoniflorin	−6.6

Docking scores are computational estimates used for descriptive ranking within each target. They do not demonstrate experimental binding or functional inhibition.

### 3.7 Quality control of GSZD

We prepared GSZD for the subsequent animal experiments by performing two successive aqueous decoctions and concentrating the combined filtrates under reduced pressure. For quality control, the resulting decoction was freeze-dried and extracted with 70% aqueous methanol, after which nine representative compounds were quantified by targeted UPLC-MS/MS in MRM mode. All nine marker compounds were successfully detected (Figures 5A–I). Timosaponin BⅡ showed the highest content (4,522.62 μg/g), followed by Trans-Cinnamic acid (2,077.09 μg/g), Paeoniflorin (1,764.51 μg/g), Neomangiferin (1,034.51 μg/g) and others (Figure 5J). Together, these results confirmed the presence of measurable marker compounds of constituent herbs, and established a marker compound profile for the prepared GSZD, providing support for the reliability of the subsequent animal experiments.

**Figure 5:**
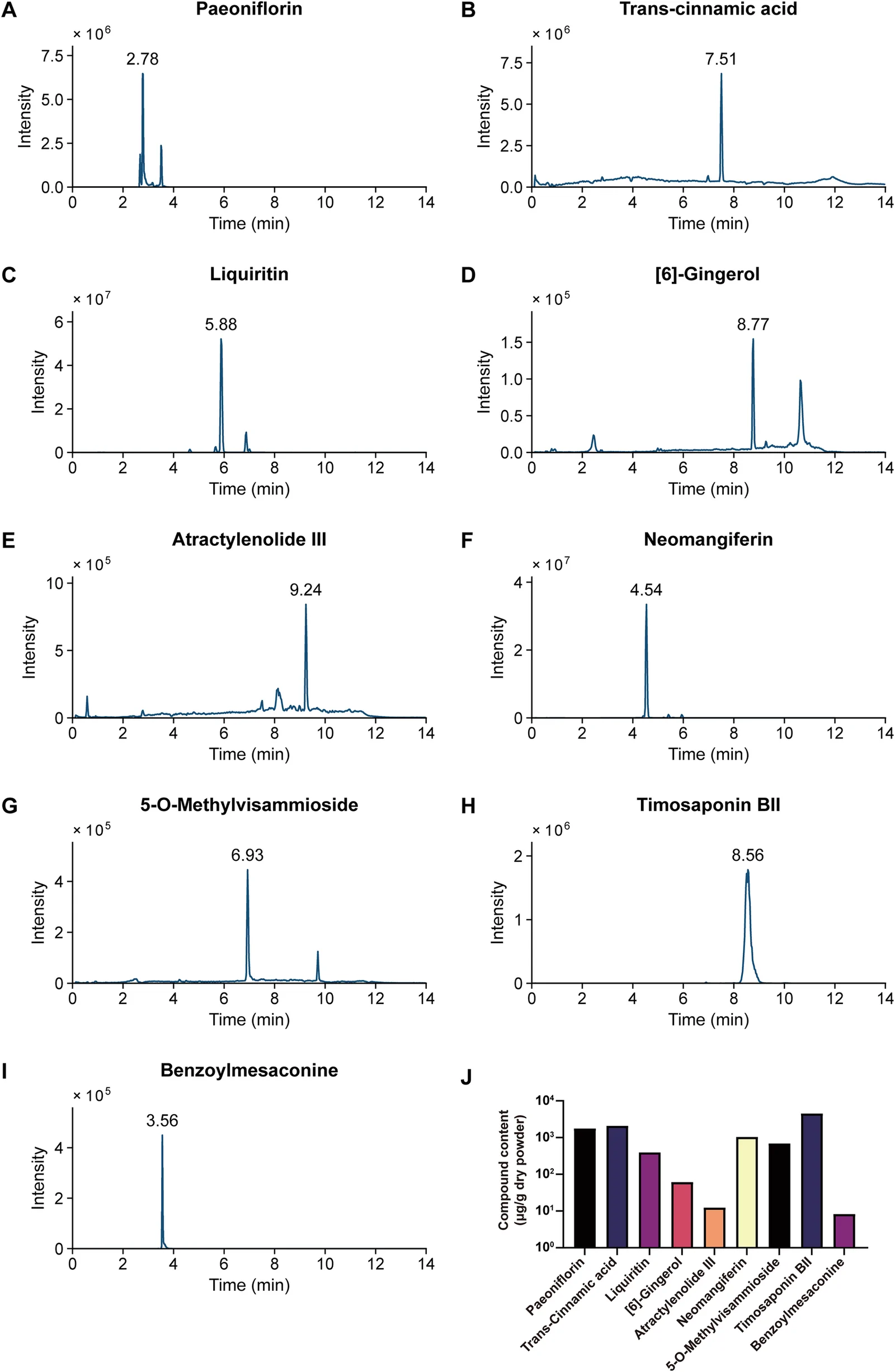
Quality control of GSZD based on UPLC-MS/MS. **(A–I)** Extracted-ion chromatograms acquired in multiple reaction monitoring (MRM) mode for Paeoniflorin **(A)**, Trans-Cinnamic acid **(B)**, Liquiritin **(C)**, [6]-Gingerol **(D)**, Atractylenolide Ⅲ **(E)**, Neomangiferin **(F)**, 5-O-Methylvisammioside **(G)**, Timosaponin BⅡ **(H)**, and Benzoylmesaconine **(I)** in the GSZD sample. Panels **A–H** were acquired in negative-ion mode, whereas panel **I** was acquired in positive-ion mode. Four repeated injections were overlaid in panel **A**. Red boxes indicated the chromatographic peaks assigned to the respective target compounds. **(J)** Contents of the nine marker compounds in lyophilized GSZD powder, expressed as μg/g dry powder. The *y*-axis was displayed on a logarithmic scale.

### 3.8 GSZD alleviated aGVHD in mouse models

Based on the preceding analysis of network pharmacology, we further performed *in vivo* experiments to verify whether GSZD could ameliorate aGVHD and corresponding gut damage. Therefore, we constructed a classical major histocompatibility complex (MHC)-mismatched aGVHD model by transplanting C57BL/6-derived bone marrow cells along with splenocytes into lethally irradiated BALB/c mice, and tested the preventive effects of GSZD administration. Our results showed that mice in the BMT group survived until day +30 after transplantation, and their body weight decreased during the first week probably because of irradiation preconditioning, followed by gradual recovery thereafter (Figures 6A,B). The mild increase in clinical scores observed in the BMT group was mainly attributable to irradiation-induced body weight loss, as a weight reduction exceeding 10% was assigned 1 point according to the scoring criteria (Figure 6C). In contrast, mice in the aGVHD group underwent rapid body weight loss without subsequent recovery, exhibited increased clinical scores, and died progressively before 3 weeks after transplantation (Figures 6A–C). In addition, mice in the aGVHD + GSZD group showed significantly prolonged survival time, compared with those in the aGVHD group (Figure 6A). Consistently, GSZD administration markedly attenuated body weight loss and reduced the clinical scores, indicating an overall alleviation of aGVHD severity (Figures 6B,C).

**Figure 6:**
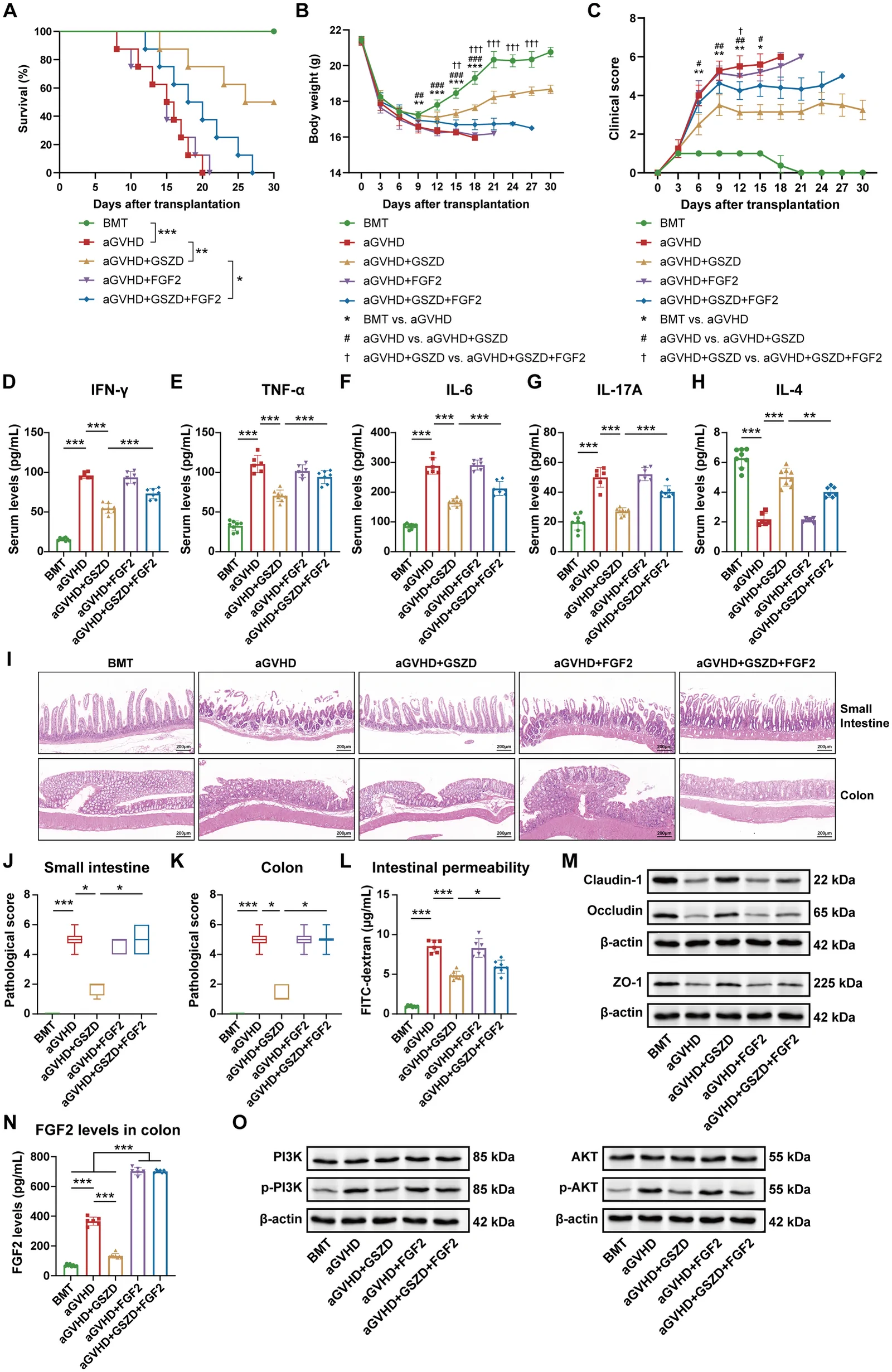
GSZD alleviated aGVHD in mouse models. **(A–C)** Survival **(A)**, body weight **(B)**, and aGVHD clinical scores **(C)** after transplantation (*n* = 8 per group). **(D–H)** Serum levels of IFN-γ **(D)**, TNF-α **(E)**, IL-6 **(F)**, IL-17A **(G)**, and IL-4 **(H)** on day +14. **(I)** Representative H&E-stained sections of the small intestine and colon. Scale bar = 200 μm. **(J,K)** Pathological scores of the small intestine **(J)** and colon **(K)**. **(L)** Intestinal permeability evaluated by serum FITC-dextran assay on day +14. **(M)** Representative Western blots of the tight junction proteins Claudin-1, Occludin, and ZO-1 (*n* = 3 per group). Densitometric results were shown in Supplementary Figures S3A–C. **(N)** FGF2 levels in colon tissues. **(O)** Representative Western blots of total and phosphorylated PI3K and AKT (*n* = 3 per group). Densitometric results were shown in Supplementary Figures S3D,E. In panels **(D–H)**, **(J–L)**, and **(N)**, surviving mice on day +14 were included for analysis (BMT: 8/8; aGVHD: 6/8; aGVHD + GSZD: 8/8; aGVHD + FGF2: 6/8; aGVHD + GSZD + FGF2: 7/8). In panels **(B)** and **(C)**, *, #, and † represented comparisons of BMT vs. aGVHD, aGVHD vs. aGVHD + GSZD, and aGVHD + GSZD vs. aGVHD + GSZD + FGF2, respectively. One, two, and three symbols indicated *P* < 0.05, *P* < 0.01, and *P* < 0.001, respectively. ns indicated no significant difference.

Cytokine storm and inflammatory activation are critical events in aGVHD initiation and development, which drive donor T cell activation and target tissue damage (18, 33). Thus, we measured serum cytokine levels and revealed that the levels of IFN-γ, TNF-α, IL-6, and IL-17A were markedly increased in the aGVHD group compared with the BMT group, whereas the Th2-associated cytokine IL-4 was decreased (Figures 6D–H). After GSZD administration, the dysregulated cytokine levels were partially restored (Figures 6D–H). Moreover, our H&E staining results showed severe pathological damage to the gut in aGVHD mice, characterized by obvious villus shortening and architectural disorder in the small intestine, together with crypt injury and mucosal structural disruption in the colon (Figures 6I–K). In contrast, mice in aGVHD + GSZD group exhibited better preservation of intestinal morphology (Figures 6I–K). Given that aGVHD-associated intestinal injury can disrupt the gut barrier, promote the translocation of luminal microbial products, and further amplify systemic inflammation, we next evaluated barrier function using a FITC-dextran permeability assay and by measuring the expression of tight junction proteins. Compared with the BMT group, the aGVHD group exhibited significantly increased intestinal permeability, accompanied by reduced expression of Claudin-1, Occludin, and ZO-1 (Figures 6L,M, Supplementary Figures S3A–C). These alterations were partially restored by GSZD, as evidenced by reduced serum FITC-dextran concentrations and elevated tight junction protein expression in the aGVHD + GSZD group (Figures 6L,M, Supplementary Figures S3A–C).

To explore the mechanism underlying these protective effects, we subsequently examined whether GSZD modulated the FGF2/PI3K/Akt signaling axis. Our ELISA analyses revealed that the FGF2 levels in the colon tissues were increased in the aGVHD group, but restored in the aGVHD + GSZD group (Figure 6N). Moreover, Western blotting results showed that the Akt and PI3K phosphorylation were elevated in the aGVHD group compared with the BMT group, while GSZD administration partially reversed these changes (Figure 6O, Supplementary Figures S3D,E).

To assess the involvement of FGF2 in GSZD-mediated protection, we also established aGVHD + FGF2 and aGVHD + GSZD + FGF2 groups, in which recombinant FGF2 was administered intrarectally. FGF2 levels in colon were significantly increased in these two groups relative to those without FGF2, confirming effective supplementation (Figure 6N). Compared with the aGVHD group, the aGVHD + FGF2 group showed no statistically significant differences in survival, body weight, clinical scores, cytokine levels, intestinal histopathology, and barrier functions, indicating that no additional worsening attributable to FGF2 was detectable under the present experimental conditions (Figures 6A–M, Supplementary Figures S3A–C). However, compared with the aGVHD + GSZD group, FGF2 supplementation in the aGVHD + GSZD + FGF2 group significantly reversed the protective effects of GSZD (Figures 6A–M, Supplementary Figures S3A–C), accompanied by reactivation of the PI3K/Akt signaling pathway (Figure 6O, Supplementary Figures S3D,E). These findings suggested that modulation of FGF2-associated PI3K/Akt signaling may contribute to the protective effects of GSZD against intestinal aGVHD.

## 4 Discussion

GI-aGVHD remains the major cause of NRM after allo-HSCT, owing to the combined effects of the alloreactive immune activation, the amplification of cytokine storm, and the gut barrier disruption (34). In this study, we integrated natural compound screening, target gene prediction, multi-database retrieval, transcriptomic WGCNA analysis, molecular docking, and *in vivo* experiment to explore the potential preventive effects of GSZD against GI-aGVHD. Our experimental results in aGVHD mouse models indicated that GSZD administration significantly prolonged survival, attenuated disease-related body weight loss and clinical or histological manifestations, and preserved the gut barrier function and the balance of cytokines. These protective effects were accompanied by reduced FGF2 levels, and decreased phosphorylation of PI3K and Akt in gut tissues, suggesting that GSZD may regulate the FGF2/PI3K/Akt signaling pathway. To our knowledge, this is the first study investigating GSZD as a potential intervention for GI-aGVHD based on network pharmacology research strategy. Overall, this study provided novel preclinical evidence which supported the preventive use of GSZD for GI-aGVHD.

GSZD was traditionally used for “Bi syndrome” in TCM practice, and had been investigated mainly in RA and other inflammatory joint disorders (15, 16, 35, 36). However, previous studies had explored new applications of several traditional formulas beyond their conventional indications, such as Banxia-Xiexin Decoction and Buyang-Huanwu Decoction, supporting the feasibility of evaluating established formulas in new disease contexts (37–40). Importantly, the reported pharmacological actions of GSZD overlap with several central inflammatory processes of aGVHD. GSZD was reported to suppress neutrophil extracellular trap formation through Cav-1/Nrf2 mechanism, inhibit IL-17R/MAPK signaling, and attenuate NF-κB/NLRP3 activation and the production of inflammatory cytokines such as IL-1β, IL-6, and MCP-1 (16, 17, 35). Meanwhile, GI-aGVHD is similarly characterized by conditioning-induced intestinal barrier damage, innate immune activation, donor T cell-driven Th1/17 responses, and increased production of cytokines including IFN-γ, TNF-α, IL-6, and IL-17A (18, 33). Intestinal barrier damage and microbial translocation can further amplify these inflammatory responses (41, 42). Thus, the existing evidence identified several biological convergence points involving the gut-immune axis, innate inflammatory amplification, and Th1/17-related signaling, providing a rationale for evaluating GSZD in GI-aGVHD. Nevertheless, RA is a chronic autoimmune disorder, whereas aGVHD is an acute alloimmune process mediated by donor-derived T cells. These shared inflammatory processes represented weak, hypothesis-generating *a priori* justification, but could not serve as independent evidence of efficacy or mechanism. Therefore, we evaluated this hypothesis by aGVHD-specific evidence: network pharmacology and gut transcriptomic analyses were used to nominate candidate targets and pathways, while animal experiments directly assessed the preventive effects of GSZD and the potential involvement of FGF2/PI3K/Akt signaling.

The blockade of a single cytokine, gene, or signaling pathway may be insufficient to control aGVHD, because of its multistage and highly interconnected pathogenesis. In contrast, GSZD, the multi-component and multi-target herbal formula, may offer broader regulation of the disease network, thereby achieving more comprehensive effects than intervention against a single molecular target. In this study, we identified 183 candidate compounds and 705 predicted target genes, indicating a broad potential pharmacological network regulated by GSZD. To further identify the subset of GSZD target genes relevant to GI-aGVHD, we needed to define the disease-associated genes. We first used multi-database retrieval, a commonly adopted strategy in network pharmacology studies, and collected 816 aGVHD-related genes. However, these genes were inherently heterogeneous, encompassing genetic susceptibility factors, inflammatory mediators, therapeutic targets, biomarkers, experimentally identified regulators, and those genes dysregulated in distinct target tissues. To address this question, we subsequently analyzed two public transcriptomic datasets from gut tissues of clinical aGVHD patients, and identified 161 genes directly associated with molecular alterations in gut tissues. Thus, database mining provided broad prior knowledge, whereas transcriptomic analysis added tissue specific evidence. Finally, we identified 109 general aGVHD target genes and 11 gut-specific aGVHD target genes, by intersecting the predicted GSZD targets with the two complementary gene sets, thereby narrowing the broad pharmacological prediction results to disease-relevant molecular networks.

In transcriptomic analysis, we applied ComBat-seq and ComBat methods to correct batch effects for count and TPM-normalized expression matrices, respectively. This step was necessary because integrating two independent datasets could introduce non-biological variations, obscuring genuine disease-related signals. We then used DESeq2 to identify DEGs, and WGCNA to identify genes with high module membership and phenotype association within co-expression modules of aGVHD. The 161 aGVHD gut damage-associated genes were identified by intersecting the DEGs with these network-associated genes. This combined strategy was adopted because differential expression analysis captured genes with significant changes in expression levels, whereas WGCNA reflected coordinated gene behavior and disease-related network structure. Their intersection enabled us to prioritize candidate genes supported by both expression dysregulation and network relevance.

To evaluate whether GSZD could alleviate GI-aGVHD through experiments, we employed the classical B6-to-BALB/c aGVHD mouse model. This fully MHC-mismatched aGVHD mouse model was widely preferred in related studies because it induced robust donor T-cell alloreactivity, typical aGVHD manifestations, and prominent gut injury (43, 44). The consistent improvements in survival, body weight, and clinical scores collectively indicated that GSZD reduced the overall severity of aGVHD. The H&E-stained pathological sections further showed that GSZD preserved villous architecture in the small intestine and crypt integrity in the colon, providing direct evidence that it attenuated gut tissue damage. Notably, the preservation of gut structures may help maintain mucosal renewal and barrier integrity, thereby limiting microbial translocation and inflammatory amplification (41, 42). In this study, the FITC-dextran assay and assessment of the tight junction proteins (Occludin, Claudin-1, and ZO-1) further confirmed increased intestinal permeability and impaired gut barrier integrity in aGVHD mice. Notably, GSZD also attenuated these barrier-related abnormalities.

The protective effects of GSZD were also accompanied by the recovery of dysregulated serum cytokines in aGVHD mice. The cytokine response in aGVHD arises from reciprocal interactions among donor T cells, antigen-presenting cells (APCs), myeloid cells, and damaged target tissues, forming an inflammatory amplification network (18). The reduction in IFN-γ after GSZD administration indicated the attenuation of excessive Th1 cell activation and cytotoxic T cell response (45). TNF-α, a central effector cytokine in aGVHD pathological process, promotes the activation of APCs, facilitates alloreactive T cell recruitment, and directly induces gut epithelial cell death and crypt damage (46). Thus, the reduction of TNF-α after GSZD administration suggested the attenuation of both systemic inflammation and tissue injury. Moreover, the decrease in IL-6 may further limit inflammatory signaling and the cytokine environment supporting pathogenic T cell differentiation, particularly Th17 responses (47). Consistently, the reduction in IL-17A, together with the significant enrichment of the term “Th17 cell differentiation” in our network pharmacology analysis, indirectly suggested that the Th17 immune axis may be involved in the biological events regulated by GSZD, although this interpretation was not directly confirmed by experiments. Furthermore, the partial restoration of IL-4 may reflect a shift toward a more balanced Th1/Th2 polarization, given the critical role of IL-4 in promoting Th2 differentiation (45). Collectively, these findings suggested that GSZD could alleviate aGVHD in mice probably by regulating dysregulated cytokine network.

As discussed above, Th1 and Th17 inflammatory responses were attenuated after GSZD administration, as evidenced by reductions in serum IFN-γ, TNF-α, and IL-17A. Although these changes contribute to the control of aGVHD, they may also compromise the beneficial graft-vs.-tumor (GVT) activity. Previous studies revealed the critical role of Th1/17 responses in antitumor immunity. Th1 responses can promote tumor cell elimination through IFN-γ/IFNGR-JAK1/2-STAT1-IRF1 signaling, which induces antigen-presentation genes, including HLA-B, HLA-C, TAP1, TAP2, and B2M, thereby enabling CD8^+^ T cell recognition and killing of malignant cells (48). In addition, Th17 responses can promote antitumor immunity through CCL20-CCR6-dependent recruitment of tumor-antigen-bearing CD8α^+^ dendritic cells that prime CD8^+^ T cells (49). However, in the allo-HSCT context, Th1/17 responses are often suppressed to control aGVHD, and such suppression may weaken GVT activity by impairing IL-17A-dependent chemokine production (such as CCL2 and CXCL1) and tumor-reactive CD8^+^ T cell recruitment, inhibiting IFN-γ/IFNGR-mediated sensitization of leukemia cells (such as the induction of MHC-Ⅰ/Ⅱ expression), and attenuating TNF-α-TNFR1-RIPK1 signaling that promotes tumor cell apoptosis and cytolysis (50–56). Taken together, GSZD-mediated inhibition of Th1/Th17 responses, although beneficial for limiting aGVHD, may adversely affect the beneficial GVT effect and thereby increase the risk of underlying malignancy relapse. Such inhibition may also compromise antimicrobial immunity. In clinical practice, infection risk during aGVHD treatment is managed with precise antimicrobial prophylaxis tailored to patients, pathogen surveillance, and preemptive treatment (57–59). Our previous study demonstrated that paeoniflorin, a representative compound of GSZD, alleviated aGVHD while preserving GVT activity, while routine blood count monitoring did not reveal hematologic abnormalities suggestive of overt infection (60). Nevertheless, the effects of the complete GSZD formulation on GVT activity and susceptibility to infection remain uncertain, and should be evaluated using murine GVT and infection models, together with flow cytometric immune profiling and cytotoxicity assays.

By integrating the results of PPI networks and functional enrichment analyses, we prioritized FGF2-associated PI3K/Akt signaling as a potential mechanism involving GSZD-mediated protection. Mechanistically, FGF2 can activate the PI3K/Akt pathway through FGFR receptors and thereby regulate a broad range of cellular processes, as reported in osteosarcoma cells, gastric cancer-associated macrophages, hair-follicle stem cells, and adipose-derived stem cells (30, 61–63). Our ELISA results showed that FGF2 levels were increased in the colon tissues of aGVHD mice. Because FGF2 can promote angiogenesis, extracellular-matrix remodeling, and tissue repair, this elevation may represent a compensatory response to ongoing intestinal damage in aGVHD development (64, 65). Increased FGF2 abundance was accompanied by increased phosphorylation of PI3K and Akt in the aGVHD group. Importantly, FGF2 supplementation in the absence of GSZD did not significantly worsen aGVHD. This may reflect a ceiling effect, as the already severe disease in the aGVHD group may have left little room for FGF2 to cause further injury. Meanwhile, FGF2 could partly reverse the protective effects of GSZD in mice treated with GSZD, accompanied by reactivation of PI3K/Akt signaling. Notably, FGF2 may promote mucosal repair in some inflammatory intestinal conditions, including experimental colitis (26). However, these protective effects of FGF2 cannot be directly extrapolated to aGVHD context, given that aGVHD differs substantially from other inflammatory intestinal diseases in underlying pathogenic mechanisms. Thus, the biological effects of FGF2 may be context-dependent. PI3K/Akt signaling is critically involved in the pathogenic process of aGVHD. For example, Pang et al. showed that excessive PI3K/Akt/mTOR activation in aGVHD patients was associated with Th1/Th17 predominance and Treg/effector T cell imbalance, while inhibiting PI3K phosphorylation attenuated IFN-γ and IL-17 production (27). In addition, Berges et al. showed that the inhibition of PI3K/Akt significantly suppressed T cell proliferation, induced cell cycle arrest and apoptosis, and favored the generation of functional Tregs (28). Collectively, our findings suggested that GSZD-mediated protection was associated with lower colonic FGF2 abundance and attenuated PI3K/Akt activation, whereas exogenous FGF2 can counteract part of this protection.

The increased endogenous FGF2 abundance observed in aGVHD prompted us to consider its potential cellular sources and review relevant studies. In normal intestine, fibroblasts, vascular endothelial cells, and resident macrophages express relatively low levels of FGF2, which supports the crypt stem/progenitor niche and epithelial renewal (66–69). During intestinal inflammation, these cell populations remain important FGF2 sources, while the release and tissue abundance of FGF2 can increase 2- to 4-fold or even more, representing a typical inflammatory stress response (69–72). For example, Shirasaka et al. showed that FGF2 was primarily expressed in fibroblasts, endothelial cells, and macrophages using immunohistochemistry, and found that the densities of FGF2^+^ cells in active UC and CD were approximately 4.6 and 4.5 times the normal level, respectively (69). In addition, Kouwenhoven et al. implicated ED2^+^ macrophages as an important source of FGF2 during intestinal allograft rejection, using RT-PCR and immunohistochemistry staining (70). FGF2^+^ cells were mainly distributed in the submucosa and muscularis, particularly around the enteric neural plexus, and their number was significantly correlated with histological injury severity (70). Furthermore, using single-cell RNA sequencing, Goto et al. found that Fgf2 was among the most strongly upregulated transcripts in intestinal RSPO3^+^GREM1^+^ fibroblasts after irradiation (71). More recently, Acharjee et al. integrated bulk and single-cell transcriptomics, and found that Fgf2 transcripts were enriched in fibrostenotic CD and were predominantly localized to fibroblasts and endothelial cells (72). However, direct evidence of the cellular source of FGF2 in aGVHD remains limited. We therefore speculate that increased endogenous FGF2 in the aGVHD gut may also arise mainly from fibroblasts, endothelial cells, and infiltrating macrophages, based on the aforementioned studies. Future studies should combine multiplex immunofluorescence and single-cell or spatial transcriptomics to provide more in-depth evidence.

Several limitations of this study should be acknowledged. First, the control groups received normal saline rather than a vehicle matched to the osmolarity, pH, and viscosity of the GSZD decoction. Although normal saline was commonly used as a gavage control in comparable animal studies, potential effects arising from these physicochemical differences could not be fully excluded (73–75). Future studies should characterize these properties and employ an appropriately validated physicochemically matched vehicle where feasible. Second, although FGF2 was delivered intrarectally and was expected to act predominantly in the colon, the pathological changes were also observed in the small intestine. This remote effect may reflect potential systemic absorption of FGF2. Previous studies have shown that several protein/peptide drugs, including high-molecular-weight urokinase, unmodified eel calcitonin, insulin, and insulin-pramlintide, exhibited systemic distribution and pharmacological effects after rectal administration (76–79), potentially via pathways such as paracellular passage between epithelial cells and vesicle-mediated transcellular endocytosis and transcytosis (80, 81). However, whether rectally administered FGF2 is systemically absorbed under aGVHD conditions remains to be determined through more in-depth experiments. In addition, the merged GEO dataset contained unequal numbers of control and aGVHD samples (18 vs. 52), owing to the limited availability of control intestinal biopsy samples. Because both WGCNA and differential expression analyses used these samples, the group imbalance may affect co-expression module construction and module-trait correlations, as well as the precision of DEG estimates. Therefore, the pronounced excess of upregulated over downregulated DEGs warranted cautious interpretation, although its cause could not be established from group sizes alone. The validation in larger, more balanced independent cohorts would further strengthen the generalizability of our results. Furthermore, the OB/DL-based screening may exclude some compounds present in the prepared GSZD that could have biological activity, leaving some potential compound-target relationships and pathways outside the network analysis. For example, Timosaponin BⅡ was the most abundant of the nine quantified marker compounds but did not meet the OB/DL criteria and was therefore excluded from downstream analyses. Nevertheless, the network analysis remains informative for the compounds that met the screening criteria, and the *in vivo* experiments evaluated the effects of the complete GSZD decoction. Finally, this study was conducted exclusively in male mice using a single C57BL/6-to-BALB/c combination and a single GSZD dose. Future dose-response studies involving female mice and other established aGVHD models are warranted to enhance the robustness and translational generalizability of our findings.

## Data Availability

The raw data supporting the conclusions of this article will be made available by the authors, without undue reservation.
