# Supplementary material for: Guizhi-Shaoyao-Zhimu decoction alleviates gastrointestinal acute graft-vs.-host disease and suppresses FGF2/PI3K/Akt signaling: insights from network pharmacology, WGCNA, molecular docking, and animal experiment
Source: Front Med (Lausanne). 2026 Oct 23;13:1908959. doi: 10.3389/fmed.2026.1908959 (PMC13739494; doi:10.3389/fmed.2026.1908959)
Supplement: Supplementary file 1 [file Datasheet2.pdf]

Supplementary Report 1

Sample Size and Power Justification Report

Manuscript: Guizhi-Shaoyao-Zhimu Decoction Alleviates Gastrointestinal Acute Graft-versus-Host Disease and Suppresses FGF2/PI3K/Akt Signaling: Insights from Network Pharmacology, WGCNA, Molecular Docking, and Animal Experiment

Authors: Xingyu Wan, Nan Li, Xueqing Yang, Wuchen Yang, Shuiqing Liu, Xi Zhang
Affiliation: Medical Center of Hematology, The Second Affiliated Hospital of Army Medical University

The required sample size was determined using G*Power version 3.1.9.7 before the conduction of animal experiments. The individual recipient mouse was set as the experimental unit. The whole study contained two parallel cohorts, and each cohort contained five experimental groups with equal allocation.

A fixed-effects omnibus one-way ANOVA was specified. Based on our preliminary experiments, the anticipated standardized effect size was set to Cohen's f = 0.60. The type I error rate was set at alpha = 0.05, and the target statistical power at 1 - beta = 0.80.

For k = 5 groups and f = 0.60, a total sample size of N = 38 provides power = 0.7971, which is below the prespecified 0.80 threshold. N = 39 is the smallest total sample size that exceeds this threshold (df1 = 4, df2 = 34, non-centrality parameter = 14.04, power = 0.8105). As equal allocation across five groups requires a total sample size divisible by five, N was rounded upward to 40. With N = 40, df1 = 4, df2 = 35, and the non-centrality parameter = 14.40, the corresponding power is 0.8232.

Conclusion: The a priori calculation therefore supported the use of 40 recipient mice per cohort, corresponding to n = 8 mice in each of the five experimental groups.

Power-analysis specification and output

Parameter	Value
Test family	F tests
Statistical test	ANOVA: Fixed effects, omnibus, one-way

Type of power analysis	A priori: Compute required sample size
Effect size	Cohen’s f = 0.60
Type I error rate	alpha = 0.05
Target power	1 - beta = 0.80
Number of groups	5
Minimum calculated total sample size	39 recipient mice
Balanced total sample size adopted	40 recipient mice
Final group size	n = 8 recipient mice per group
Power at N = 40	0.8232 (82.3%)

Reference: Faul F, Erdfelder E, Buchner A, Lang AG. Statistical power analyses using G*Power 3.1: tests for correlation and regression analyses. Behav Res Methods. 2009;41:1149-1160. doi:10.3758/BRM.41.4.1149.
