# Supplementary material for: Guizhi-Shaoyao-Zhimu decoction alleviates gastrointestinal acute graft-vs.-host disease and suppresses FGF2/PI3K/Akt signaling: insights from network pharmacology, WGCNA, molecular docking, and animal experiment
Source: Front Med (Lausanne). 2026 Oct 23;13:1908959. doi: 10.3389/fmed.2026.1908959 (PMC13739494; doi:10.3389/fmed.2026.1908959)
Supplement: Supplementary file 2 [file Datasheet1.docx]

**Supplementary Table 1. Search strategy for genes related to aGVHD**

| **Databases** | **Search strategy** |
| --- | --- |
| GeneCards (https://www.genecards.org/) | The term “acute graft-versus-host disease” was searched, and all related targets were exported. |
| OMIM (https://www.omim.org/) | Using the “Gene Map” function under “Advanced Search”, the term “acute graft-versus-host disease” was searched, and all related targets were exported. |
| DrugBank (https://go.drugbank.com/) | Under the “Indications” option, the term “acute graft-versus-host disease” was searched. The matching disease term (ID: DBCOND0125377) was then selected, and all related targets were exported. |
| PharmGKB (https://www.clinpgx.org/) | The term “acute graft-versus-host disease” was searched. The matching disease term (ID: PA444320) was then selected, and all related targets were exported. |
| SymMap (http://www.symmap.org/) | Under the “Disease” option, the term “Graft-Vs-Host Disease” was searched. The matching disease term (ID: SMDE09127) was then selected, and all related targets were exported. |
| TTD (https://ttd.idrblab.cn/) | Under the “Search Target or Drug by ICD-11 Defined Disease Class” option, the term “Graft-versus-host disease” was searched. The matching disease term (ID: 4B24) was then selected, and all related targets were exported. |
| HERB (http://herb.ac.cn/) | Under the “Disease” option, the term “Graft-vs-Host Disease” was searched. The matching disease term (ID: HBDIS001214) was then selected, and all related targets were exported. |

**Supplementary Table 2. Herbal composition and dosage of GSZD**

| **Chinese name** | **Latin name** | **Weight** |
| --- | --- | --- |
| Guizhi | Cinnamomi Ramulus | 120 g |
| Mahuang | Ephedrae Herba | 120 g |
| Fuzi | Aconiti Lateralis Radix Praeparata | 100 g |
| Zhimu | Anemarrhenae Rhizoma | 120 g |
| Baishao | Paeoniae Radix Alba | 90 g |
| Baizhu | Atractylodis Macrocephalae Rhizoma | 150 g |
| Fangfeng | Saposhnikoviae Radix | 120 g |
| Shengjiang | Zingiberis Rhizoma Recens | 150 g |
| Gancao | Glycyrrhizae Radix et Rhizoma | 60 g |
| **Total** | | 1030 g |

**Supplementary Table 3. Batch correction efficacy metrics**

| **Metric** | **Before correction** | **After correction** |
| --- | --- | --- |
| Silhouette coefficient (batch) | 0.5745 | 0.0442 |
| Silhouette coefficient (biological groups) | 0.0909 | 0.0832 |
| PERMANOVA R^2^ (batch) | 0.4683 | 0.0095 |
| PERMANOVA p (batch) | 0.002 (**) | 0.586 |
| PERMANOVA R^2^ (biological groups) | 0.0381 | 0.1099 |
| PERMANOVA p (biological groups) | 0.046 (*) | 0.002 (**) |

* P <0.05 ** P < 0.01

**Supplementary Table 4. The information for aGVHD-associated modules**

| **Module** | **Total genes** | **Correlation with aGVHD** | **P** | **Genes meeting MM/GS criteria** |
| --- | --- | --- | --- | --- |
| Darkgrey | 49 | -0.49497 | 1.3253E-05 | 4 |
| Black | 7,038 | 0.36887 | 1.6767E-03 | 566 |
| Darkgreen | 4,033 | 0.54202 | 1.2593E-06 | 732 |
| Darkturquoise | 258 | 0.35046 | 2.9363E-03 | 10 |

**Supplementary Table 5. Sensitivity analysis of the PPI network using medium- and high-confidence STRING thresholds**

| **Characteristic** | **Medium confidence (confidence ≥ 0.400)** | **High confidence (confidence ≥ 0.700)** | **Sensitivity result** |
| --- | --- | --- | --- |
| Input genes | 109 | 109 | —— |
| Network nodes (≥1 edge) | 105 | 95 | 95/105 retained (90.5%) |
| Network edges | 1,474 | 507 | 507/1,474 retained (34.4%) |
| Top 10 genes ranked by degree | IL6, TNF, IL1B, AKT1, STAT3, PIK3CD, MTOR, NFKB1, TLR4, BCL2 | IL6, TNF, IL1B, STAT3, AKT1, NFKB1, PIK3CD, EGFR, STAT1, BCL2 | 8/10 retained  (Jaccard index = 0.667) |
| Overall degree-rank concordance | —— | —— | Spearman’s ρ = 0.912; P < 0.001 |

**Note**: The same 109 input genes were evaluated at two different STRING confidence thresholds. Network nodes refer to genes appearing in at least one interaction edge. The Top 10 genes were ranked by degree centrality. Spearman correlation was calculated across the 105 gene nodes in the 0.400 edge file, with genes absent from the 0.700 edge file assigned a degree of zero.

**Supplementary Figure 1. Visualization of transcriptomic data before and after batch effect correction.**

(A) PCA plots showing sample distribution before and after correction. (B) Boxplots showing the distribution of gene expression before and after correction. (C) Clustering heatmap showing the gene expression of the top 50 genes most strongly associated with batch effects before and after correction.

**Supplementary Figure 2. Sankey and bubble plots of GO and KEGG enrichment analyses of shared genes between Gene List A and C.**

Sankey links connected genes to the corresponding GO terms and KEGG pathways. Bubble size indicated gene count, color represented -log_10_(P), and the horizontal axis showed the gene ratio (%).

**Supplementary Figure 3. Quantification of Western blot results.**

Colon tissues were collected on day +14 after transplantation from 3 randomly selected mice per group in Cohort 2. (A-C) Relative protein levels of Claudin-1 (A), Occludin (B), and ZO-1 (C), normalized to β-actin. (D-E) Phosphorylation levels of AKT (D) and PI3K (E), normalized to total AKT and PI3K. * P < 0.05, ** P < 0.01, *** P < 0.001.
